# Remarks of Dr. A. A. McKittrick at the American Anti-Tuberculosis League, Recently Convened in Atlanta, Ga.

**Published:** 1905-06

**Authors:** 


### REMARKS OF DR. A. A. McKITTRICK AT THE AMERICAN ANTI-TUBERCULOSIS LEAGUE, RECENTLY CONVENED IN ATLANTA, GEORGIA.

MR. PRESIDENT: I have been unable to prepare the paper proposed—"Differential Diagnosis of Tuberculosis and Some Lung Diseases," my eyes being affected with optical neuritis.

Having had experience in the treatment of lung diseases, I will make a few practical remarks as to them, excluding pathology, microscopy and symptomatology, only asserting a climacteric and detergent influence in preventing and ameliorating tuberculosis.

I have been residing at Evergreen, Conecuh County, Alabama, nearly forty years. It is situated on the Louisville & Nashville Railroad about midway between Montgomery and Mobile, Alabama, twenty-five miles from Florida and seventy-five miles from the Gulf of Mexico, whose gentle and balmy breezes are wafted to us; its level above tide water being 287 feet at Evergreen, varying from 250 to 385 feet, having a rolling and undulating surface with an argillaceous and sandy soil, abundantly and beautifully interspersed with pines, gifted with sanitary virtues in every bough, emitting ozone, a powerful disinfectant and purifier of the atmosphere; deserves its unsurpassed reputation for salubrity.

As a favorable residence for consumptives, it may justly claim to have superior advantages over Florida, its atmosphere being neither too moist nor dry. In proof of the fact, many persons can be cited who came here from the North and elsewhere with hemorrhages from their lungs, who by a continued residence have permanently recovered. Many are seen here daily whose lives have been prolonged. People from the North and West have made Evergreen, Alabama, a favorable resort for health. Many affected with tuberculosis, and invalids, have returned to their homes restored.

The sanitary status of Conecuh County is extraordinary, proved by statistics that can be vouched for as reliable. *Our late greatly lamented State Health Officer,* Dr. Jerome Cochran, continually doubted from year to year the correctness of our low death-rate. It was verified and he said, "The healthiest country the sun shines on never had such a small death-rate." Our doctors and people are in position to know the fact, and will testify that the statistics are accurate.

Prior to our Civil War negroes were unaffected as now with lung diseases. They are being depopulated by what is termed consumption. Doubtless this is caused by a change of their environments. Their freedom has caused them to endanger themselves by improvident care in various ways, resulting mostly in lung affections. There is certainly a difference in the anatomical structure of their lungs from those of the white. They endure with much less impunity exposure.

A great many whites die from sequelæ of measles, their lungs being affected with symptoms of consumption. A true diagnosis of these cases is not tuberculosis. Also many of lymphatic temperament, whose lungs sympathize with derangement of the liver, containing no tubercles, die with pthisis pulmonalis. As to treatment, I rely not so much on medicine as sanitary environments—pure air, dietetics and good nursing. I have found petroleum and creosote to be the best medicine. We are groping in the dark about consumption. There are constant developments and discoveries. Since my arrival in Atlanta I have heard of the paper of Dr. Curtis from Wilcox County, Alabama, favorably commented, advocating the inoculative treatment with the watery extract from the sputa of tuberculosis patients. I do not endorse it. I suppose he grounded his theory on the idea that one poison counteracts another. I have heard of the tenae or tapeworm preventing tuberculosis. The late Dr. Edmund Gaines, of Mobile, Alabama, lost one lung by consumption, and he told us that he was willing to sacrifice his life for the benefit of science to prove that if his fistula in ano were cured, he would very soon die perhaps. His life was prolonged according to the aphorism of Hippocrates "*Ubi irritatio ibi fluxus*"—wherever there is an irritation, there is a flow.

Mr. President, in laudation of Evergreen, Alabama, please allow me a digression. Like this great city of Atlanta, it does not afford a nidus for the yellow fever virus. During the sixties, seventies and eighties, cases of yellow fever were brought to Evergreen. They were nursed, people came in contact with them, and yellow fever did not spread. I inhaled the air from the lungs of the second patient with yellow fever, at Brewton, Alabama. Leaving immediately after the death, I believe the air of Evergreen prevented me from contracting it. The corpse of the first patient at Brewton was transported through Evergreen, remaining in our depot all night; the lid of the coffin pressed open and odors emanated from it. It was carried to the country for burial; people were exposed to it, and yellow fever did not spread. Our climate does not only prevent tuberculosis, but also yellow fever.

